# Biomechanical characteristics of fixation methods for floating pubic symphysis

**DOI:** 10.1186/s13018-017-0541-z

**Published:** 2017-03-07

**Authors:** Wenhao Song, Dongsheng Zhou, Yu He

**Affiliations:** Department of Orthopedic Surgery, Shandong Provincial Hospital Affiliated to Shandong University, 324 Jingwu Road, Ji’nan, Shandong People’s Republic of China

**Keywords:** Floating pubic symphysis, Biomechanical characteristics, External fixation, Subcutaneous fixation, Internal fixation

## Abstract

**Background:**

Floating pubic symphysis (FPS) is a relatively rare injury caused by high-energy mechanisms. There are several fixation methods used to treat FPS, including external fixation, subcutaneous fixation, internal fixation, and percutaneous cannulated screw fixation. To choose the appropriate fixation, it is necessary to study the biomechanical performance of these different methods. The goal of this study was to compare the biomechanical characteristics of six methods by finite element analysis.

**Methods:**

A three-dimensional finite element model of FPS was simulated. Six methods were used in the FPS model, including external fixation (Ext), subcutaneous rod fixation (Sub-rod), subcutaneous plate fixation (Sub-plate), superior pectineal plate fixation (Int-sup), infrapectineal plate fixation (Int-ifa), and cannulated screw fixation (Int-scr). Compressive and rotational loads were then applied in all models. Biomechanical characteristics that were recorded and analyzed included construct stiffness, micromotion of the fracture gaps, von Mises stress, and stress distribution.

**Results:**

The construct stiffness of the anterior pelvic ring was decreased dramatically when FPS occurred. Compressive stiffness was restored by the three internal fixation and Sub-rod methods. Unfortunately, rotational stiffness was not restored satisfactorily by the six methods. For micromotion of the fracture gaps, the displacement was reduced significantly by the Int-sup and Int-ifa methods under compression. The internal fixation methods and Sub-plate method performed well under rotation. The maximum von Mises stress of the implants was not large. For the plate-screw system, the maximum von Mises stress occurred over the region of the fracture and plate-screw joints. The maximum von Mises stress appeared on the rod-screw and screw-bone interfaces for the rod-screw system.

**Conclusions:**

The present study showed the biomechanical advantages of internal fixation methods for FPS from a finite element view. Superior stabilization of the anterior pelvic ring and fracture gaps was obtained by internal fixation. Subcutaneous fixation had satisfactory outcomes as well. Sub-rod fixation offered good anti-compression, while the Sub-plate fixation provided favorable anti-rotational capacity.

## Background

Floating injuries have been discussed frequently in the literature and describe a unique fracture pattern; examples include floating shoulder [1], floating hip [2], and floating knee [3]. A floating joint is one that has lost its continuity at adjacent ends and has no bony attachments on either end [4]. The pubic symphysis is an oligodynamic joint and consists of bilateral pubic bones and a fibrocartilaginous disc. Fractures of the bilateral superior and inferior pubic rami and ischial rami are considered to create a floating pubic symphysis (FPS). In this situation, the pubic symphysis has lost its continuity with the innominate bones and the anterior pelvic ring has become extremely unstable.

FPS is a relatively rare injury caused by high-energy mechanisms [3, 4] and causes a disruption in the normal biomechanical function of the anterior pelvic ring. Because the risk of hemorrhagic shock and rectal, urogenital, and vaginal injuries increases dramatically [5], the mechanical and architectural stability of the anterior pelvic ring must be restored.

Depending on the energy level of the trauma, the mortality rate is between 18 and 25% in patients with hemodynamic instability [6]. Therefore, pathophysiological and hemodynamic stabilization should be considered carefully before surgical intervention is undertaken. For patients with hemodynamic instability, maneuvers should be performed to decrease pelvic volume and reduce motion of the bony fragments. The aim of these early damage control techniques is to achieve relative stability in a minimally invasive manner [7–10]. For patients who are hemodynamically stable, early definitive fixation can be undertaken with the goals being good functional recovery and a return to normal life. The purpose of definitive fixation is accurate reduction, rigid fixation, and minimal soft tissue disruption. There are several fixation methods used to treat FPS, including external fixation [11], anterior subcutaneous fixation [10, 12], internal fixation [13], and percutaneous cannulated screw fixation [14]. To choose the optimal fixation method, it is necessary to study the biomechanical performance of the different methods.

Therefore, the purpose of this study was to compare the biomechanical characteristics of six fixation methods for FPS using finite element analysis. Fixation methods were divided into three groups: external, subcutaneous, and internal fixation. In the subcutaneous group, there were two methods, subcutaneous rod and subcutaneous plate fixation. The superior pectineal plate (the ilioinguinal approach), infrapectineal plate (the modified Stoppa approach), and cannulated screw fixation were methods in the internal fixation group.

## Methods

### Finite element models and implants

**Fig. 1:**
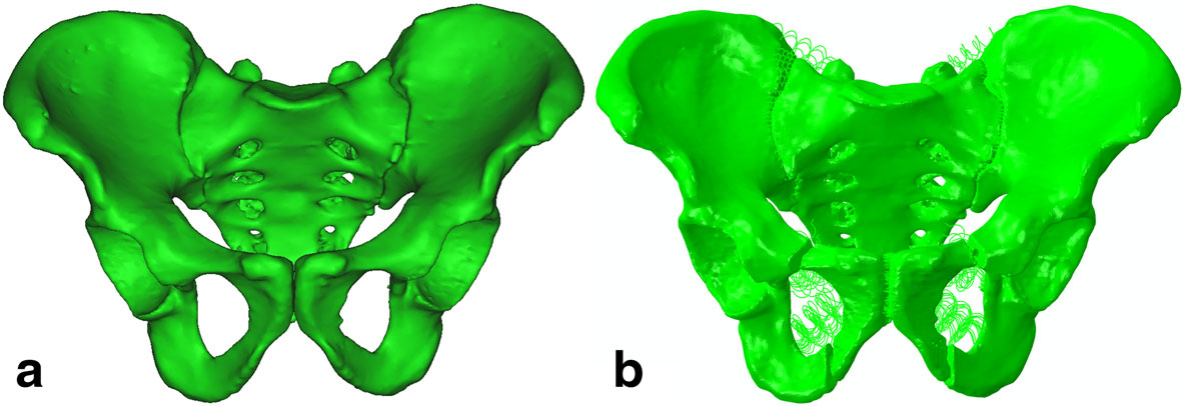
The intact (**a**) and floating pubic symphysis (**b**) models

The three-dimensional finite element model was obtained from the database of Shandong Provincial Hospital Affiliated to Shandong University. The reliability and validity was measured strictly by biomechanics laboratory. The model developed from 0.65-mm thin-section computed tomography (Lightspeed VCT, GE, Fairfield, CT) scans of a healthy volunteer (178 cm, 63 kg, 35 years old, male). The values of window width, rows, and columns were 250, 512, and 512, respectively. The DICOM format files of CT scans were processed using MIMICS 15.0 (Materialise, Belgium), and a bony pelvic model was created. The anterior sacroiliac, posterior sacroiliac, interosseous sacroiliac, sacrospinous, sacrotuberous, superior pubic, and arcuate pubic ligaments were added to the bony model in Abaqus 6.13 (3DS, Waltham, MA, USA) to simulate the normal skeleton-ligament system (Fig. 1a). The ligaments were simulated as non-linear spring elements. To simulate FPS (Fig. 1b), the model included 5-mm bone defects in the bilateral superior and inferior pubic rami.

**Fig. 2:**
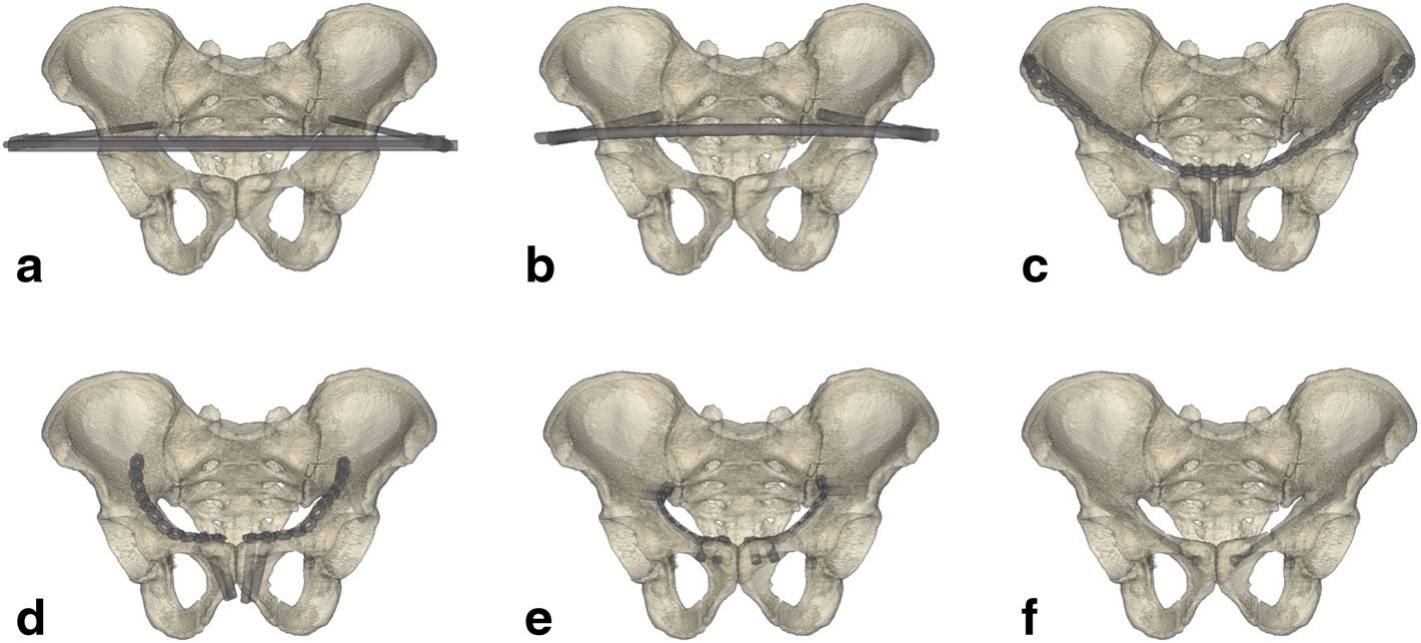
The external fixation (**a**), subcutaneous rod fixation (**b**), subcutaneous plate fixation (**c**), superior pectineal plate fixation (**d**), infrapectineal plate fixation (**e**), and cannulated screw fixation (**f**) were used in the floating pubic symphysis model

Six fixation methods were used in the FPS model, including external fixation (Ext), subcutaneous rod fixation (Sub-rod), subcutaneous plate fixation (Sub-plate), superior pectineal plate fixation (Int-sup), infrapectineal plate fixation (Int-ifa), and cannulated screw fixation (Int-scr) (Fig. 2). The external fixation apparatus (Constant, Wuhan, China) was mounted using two 5-mm apex self-drilling pins and two 8-mm connecting rods. The diameters of the pedicle screws (Medtronic-WeiGao Inc., WeiHai, China) and connecting rod (Constant, Wuhan, China) in the Sub-rod fixation system were 8 and 8.5 mm, respectively. The reconstruction plates (Synthes, Oberdorf, Switzerland) were 17-hole (x2) for the Sub-plate, 11-hole (x2) for the Int-sup, and 9-hole (x2) for the Int-ifa methods, respectively. The length and diameter of the cannulated screws (Synthes, Oberdorf, Switzerland) were 100 and 7.3 mm, respectively. All implants were inserted into the FPS model using a standard surgical technique. The contact behavior of the screw-bone interface was set as a rigid bond and that of the plate-bone interface as surface-to-surface. The threads and screw heads of the cannulated screws were fully fixed into bones. The threads of the cortical screws were omitted to simplify the models.

The intact model without implants had a total of 769,120 elements and 204,680 nodes. The numbers of elements for implants were 86,682, 59,305, 198,675, 158,372, 123,885, and 59,168 for the Ext, Sub-rod, Sub-plate, Int-sup, Int-ifa, and Int-scr methods, respectively. The numbers of nodes for these implants were 20,696, 14,095, 47,543, 37,153, 28,307, and 16,332, respectively.

### Finite element analysis

The finite element analysis was performed using Abaqus 6.13. Linear elastic isotropic material properties were assigned to all models and implants. All contact elements were defined as deformable elements. The properties of the bones, ligaments, and implants [15] are shown in Table 1.

**Table 1:** Material properties of finite element models

Material	Elastic modulus (MPa)	Poisson’s ratio	*K* (N/m)	Number of springs
Bone				
Cortical bone	18,000	0.3		
Cancellous bone	150	0.2		
Ligaments				
Anterior and capsule sacroiliac ligament			700	27
Posterior sacroiliac ligament			1400	15
Interosseous sacroiliac ligament			2800	8
Sacrospinous ligament			1400	9
Sacrotuberous ligament			1500	15
Superior pubic ligament			500	24
Arcuate pubic ligament			500	24
Implants	114,000	0.3		

**Fig. 3:**
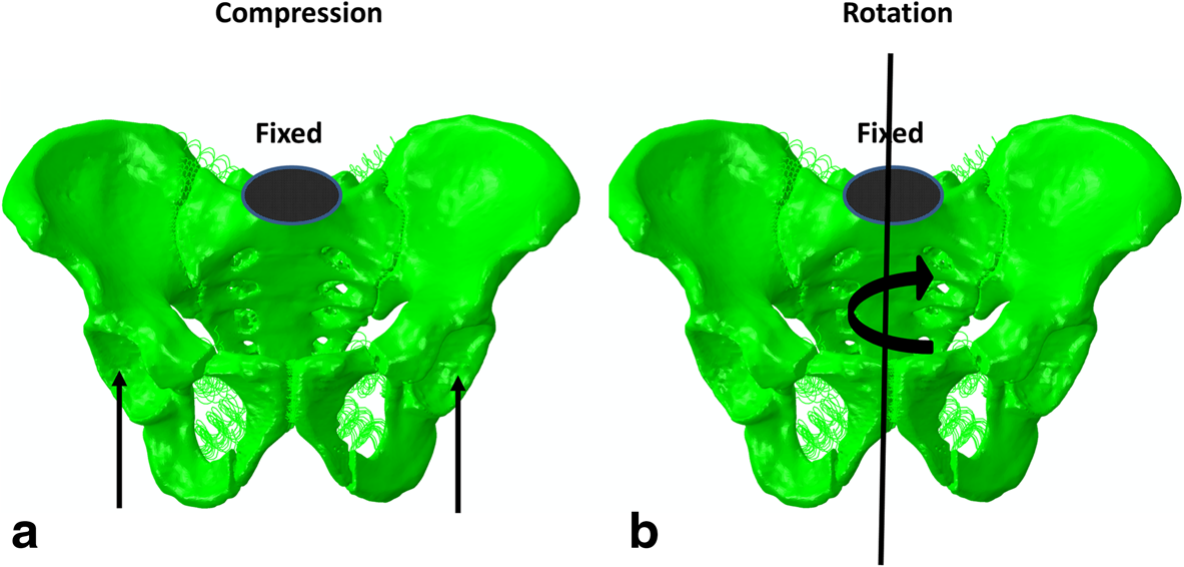
The load conditions of finite element analysis. The superior segment of S1 of the models was fixed. Two types of load condition were used in all models, including compression (**a**) and rotation (**b**)

The superior segment of S1 in the models was fixed. The two types of load conditions that were used in all models were compression and rotation (Fig. 3). For compression, the vertical force of 250 N was distributed to each acetabulum. For rotation, a torque of 7 Nm was applied to the pelvis to simulate rotation.

The biomechanical characteristics of the Ext, Sub-rod, Sub-plate, Int-sup, Int-ifa, and Int-scr models were analyzed and compared using the normal and FPS models. Construct stiffness was used to compare anterior pelvic ring stability. The stabilization of the fracture regions was evaluated by recording the increments of micromotion of the fracture gaps. To evaluate the forces, stress distribution and maximum von Mises stress were measured.

## Results

The construct stiffness of the different models under compression and rotation is displayed in Table 2. When FPS occurred, the construct stiffness of the anterior pelvic ring was decreased dramatically under both conditions. The compressive construct stiffness of FPS fixation using the Sub-rod, Int-sup, Int-ifa, and Int-scr methods were 61.495, 97.504, 105.530, 104.280, and 90.469% of that seen in the normal model, respectively. In general, the compressive stiffness was restored by the internal fixation and Sub-rod methods. Unfortunately, rotational stiffness was not restored satisfactorily by the six methods. The method that best restored the rotational stiffness was the Int-ifa method, and the stiffness was only 75.6% of that seen in the normal model.

**Table 2:** The construct stiffness of models

Load condition	Nor	FPS	Ext	Sub-rod	Sub-plate	Int-sup	Int-ifa	Int-scr
Compression (%)	100	61.49533	81.33454	97.50432	76.74699	105.5299	104.2795	90.46921
Rotation (%)	100	42.34384	42.83913	43.98612	63.57524	69.24526	75.61063	70.69581

The incremental micromotion of the fracture region is shown in Table 3. The FPS model showed extreme instability. Under compressive loads, the displacement was reduced significantly by the internal fixation systems. The Int-sup and Int-ifa methods were the most effective, with displacement of only 0.068 and 0.045 mm, respectively. Under rotational loads, the internal fixation systems and Sub-plate performed well with 0.048°, 0.056°, 0.005°, and 0.150° of fracture region micromotion using the Int-sup, Int-ifa, Int-scr, and Sub-plate methods, respectively.

**Table 3:** The incremental micromotion of fracture region

Load condition	FPS	Ext	Sub-rod	Sub-plate	Int-sup	Int-ifa	Int-scr
Compression (mm)	0.722	0.552	0.462	0.725	0.068	0.045	0.304
Rotation (°)	1.565	1.559	1.534	0.150	0.048	0.056	0.005

**Fig. 4:**
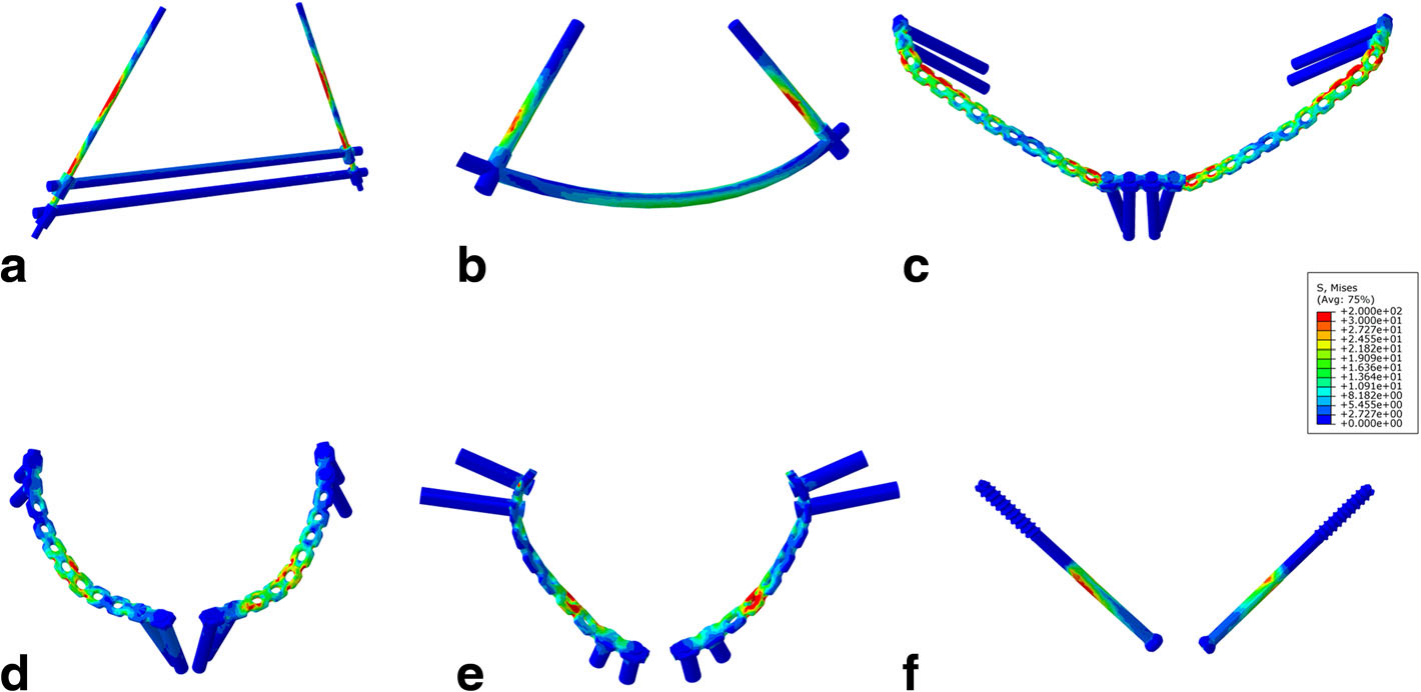
The von Misses stress of external fixation (**a**), subcutaneous rod fixation (**b**), subcutaneous plate fixation (**c**), superior pectineal plate fixation (**d**), infrapectineal plate fixation (**e**), and cannulated screw fixation (**f**) under compressional load condition

**Fig. 5:**
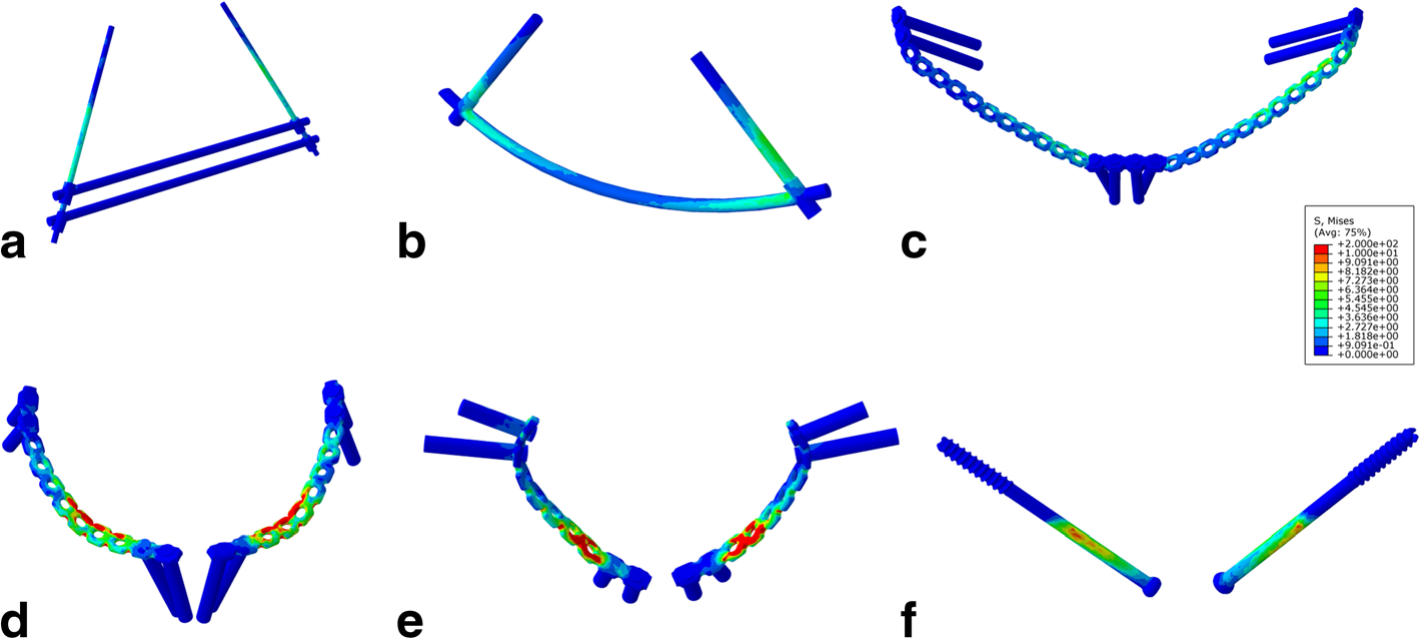
The von Misses stress of external fixation (**a**), subcutaneous rod fixation (**b**), subcutaneous plate fixation (**c**), superior pectineal plate fixation (**d**), infrapectineal plate fixation (**e**), and cannulated screw fixation (**f**) under rotational load condition

The von Mises stress of the implants is shown in Figs. 4 and 5 and Table 4. In general, the maximum von Mises stress of the implants was not large. The Sub-plate and Int-scr endured the highest level of stress. For the plate-screw system, the maximum von Mises stress occurred over the region of the fracture and the plate-screw joints. With respect to the reconstruction plate, the molding sites, especially for the Sub-plate, endured the maximum von Mises stress. In the rod-screw system, the maximum von Mises stress appeared at the rod-screw and screw-bone interfaces.

**Table 4:** The maximum von Misses stress of implants

Load condition	Ext	Sub-rod	Sub-plate	Int-sup	Int-ifa	Int-scr
Compression (MPa)	133.148	108.481	214.616	165.828	126.683	218.38
Rotation (MPa)	12.536	9.452	25.739	61.783	62.87	30.343

In summary, the level of stability of the anterior pelvic ring and fracture regions was highest using the internal fixation methods, followed by the subcutaneous fixation methods, and lowest using external fixation. Interestingly, the Sub-rod and Sub-plate showed completely different characteristics. The Sub-rod offered satisfactory anti-compression, while the Sub-plate provided better anti-rotational capacity.

## Discussion

FPS is a life-threatening injury characterized by an unstable anterior pelvic ring and hemodynamic instability. The instability of the pelvic ring is caused by fractures of the bilateral superior and inferior pubic rami and ischial rami. The mortality rate is 8.5–19% in polytrauma patients with pelvic fractures [6]. Shockingly, the mortality rate increases to 18–25% in patients with hemodynamic instability [6]. Moreover, the risk of internal organ injuries increases when there are migrating fracture fragments [5]. Thus, patients with FPS require emergency pelvic stabilization to help minimize the mortality rate. Several fixation methods have been applied under such circumstances, including external fixation, anterior subcutaneous fixation, internal plate fixation, and percutaneous cannulated screw fixation. To choose the optimal fixation method, it is necessary to study the biomechanical performance of the different methods. Therefore, the aim of this study was to compare the biomechanical characteristics of six methods using finite element analysis.

External fixation remains the gold standard in the emergent treatment of anterior pelvic fractures [6], especially in patients with hemorrhagic shock and/or significant soft tissue damage. However, there are disadvantages to using an external fixator, including pin tract infection, loosening, skin impingement by pins, osteomyelitis, inconvenience regarding wound care and mobility, and negative effects on a patient’s quality of life [16, 17]. Particularly in diabetic and obese patients, external fixation is not recommended [16, 18]. External fixation can be a definitive treatment when accurate reduction and adequate fixation is obtained. Unfortunately, our results showed that external fixation did not meet these criteria from a biomechanical point of view. Under both compressive and rotational loads, the construct stiffness of the anterior pelvic ring was the lowest with external fixation. Failure to achieve and maintain stability of the fracture fragments can lead to recurrent bleeding and secondary internal organ injuries. Post-operative fragment dislocation is also a result of deficient fixation, and there are multiple secondary injuries that can be caused by unstable fracture fragments. Therefore, methods achieving increased construct stiffness may be necessary for definitive treatment after emergent stabilization has been performed.

We applied two techniques for achieving subcutaneous anterior fixation, including the use of a rod [18] and a plate [12]. These techniques have been performed in several cases. According to those who developed these methods [10, 17], subcutaneous fixation is appropriate in patients with hemodynamic instability. Operative time is comparable to that seen with external fixation, and the complication rate was reported to be lower. However, different complications including irritation of the lateral femoral cutaneous nerve and heterotopic ossification formation were shown to occur with subcutaneous rod fixation. The plate is not easy to apply in the setting of FPS due to difficulty in achieving reduction. From a biomechanical perspective, our results showed that subcutaneous anterior fixation achieves greater stiffness than external fixation. Interestingly, the subcutaneous rod fixator responded well to compression, while the subcutaneous plate fixator provided favorable anti-rotational capacity. This may be because the rod is ilium-ilium fixation and the plate is ilium-pubis-ilium fixation.

With regard to internal plate fixation methods, our study showed no significant difference between the use of the superior pectineal plate and infrapectineal plate. Both provided adequate stability from a biomechanical perspective. Indications for fixation methods depend on the fracture pattern and should be an area of future clinical research. Cannulated screws are an alternative option that have had good outcomes with use in FPS. This technique avoids extensive surgical exposure and minimizes the complications often experienced with reconstruction plates [14]. Furthermore, cannulated screws can achieve similar biomechanical results to internal fixation. Percutaneous cannulated screw fixation can provide accurate closed reduction and rigid fixation and is an excellent option for the treatment of patients with hemodynamic instability and soft tissue damage [14]. This technique can be performed in both emergent and delayed stabilization. Reduced operative times and diminished blood loss are advantages of percutaneous cannulated screw fixation which help to contribute to lower complication rates. However, frequent intra-operative fluoroscopy using a navigation system is required for accurate screw insertion.

Additionally, it should be mentioned that FPS is often associated with posterior pelvic ring injuries that cannot be restored and stabilized using the fixation methods previously described. Posterior pelvic ring stabilization can be achieved by percutaneous sacroiliac screws or other methods. In the present study, we maintained an intact posterior pelvic ring to minimize confounding factors. This was a limitation of our research.

There were other limitations of this study. The finite element models were based on a skeleton-ligament system, and thus, the muscle forces were not accounted for. This is similar to other finite element studies. A single pelvic model was used for analysis, which may neglect the bony and ligamentous variation seen between people. In the current study, we used the Chinese external fixation system, Constant. Considering that different implants have disparate designs, the results of this research can only be generalized to similar external fixators. The present study was designed to evaluate the biomechanical characteristics of six methods in the treatment of FPS, but pathophysiological and hemodynamic stabilization was not considered. Additional aspects of surgical treatment should be addressed in future clinical studies.

## Conclusions

In conclusion, from the finite element view, the present study showed the biomechanical advantages of internal fixation for FPS compared with external fixation. Improved stabilization of the anterior pelvic ring and fracture gaps was obtained using internal fixation. Subcutaneous fixation had satisfactory outcomes as well. The Sub-rod fixation offered good anti-compression, while Sub-plate fixation provided favorable anti-rotational capacity.

## Abbreviations

CT: Computed tomography
Ext: External fixation
FPS: Floating pubic symphysis
Int-ifa: Infrapectineal plate fixation
Int-scr: Cannulated screw fixation
Int-sup: Superior pectineal plate fixation
Sub-plate: Subcutaneous plate fixation
Sub-rod: Subcutaneous rod fixation
